# Knowledge, attitude and practice of emergency care providers on obstetric haemorrhage in KwaZulu-Natal, South Africa: A cross-sectional study

**DOI:** 10.1016/j.afjem.2025.100909

**Published:** 2025-09-27

**Authors:** S Govender, OP Khaliq, T Abel, J Moodley

**Affiliations:** a KwaZulu-Natal College of Emergency Care, Durban, South Africa; b Women’s Health and HIV Research Group, Department of Obstetrics and Gynaecology, School of Medicine, College of Health Sciences, University of KwaZulu-Natal, Durban, South Africa; c Department of Paediatrics and Child Health, Faculty of Health Sciences, School of Clinical Medicine, University of the Free State, Bloemfontein, South Africa; d Department of Otorhinolaryngology, School of Medicine, College of Health Sciences, University of KwaZulu-Natal, Durban, South Africa

**Keywords:** Emergency medical service, Emergency care providers, Obstetric haemorrhage, KwaZulu-Natal

## Abstract

**Background:**

Obstetric haemorrhage (OH) is a leading cause of maternal deaths worldwide. The South African Department of Health recommends that all childbirths be managed by skilled personnel who can identify and manage complications to reduce adverse maternal, perinatal and neonatal outcomes. Emergency Care (EC) providers are prehospital personnel mandated to fulfil this role; however, little is known about their knowledge, attitudes and practices (KAP) for OH emergencies. This study assesses EC providers’ knowledge (specifically pathophysiology, diagnosis and treatment), attitude and practices regarding OH management in KwaZulu-Natal (KZN), South Africa.

**Methods:**

A cross-sectional KAP survey was administered to public-sector EC providers at designated central locations in each of the 11 KZN districts, with participants representing multiple ambulance stations in their respective districts. Data were collected through a structured questionnaire completed by 417 participants enrolled between July 2022 and August 2023. Modified Bloom’s cut-off points were used to develop KAP scores, enabling categorical assessment. Data analysis included descriptive and inferential statistics.

**Results:**

Participants (*n* = 417) were predominantly male (75.3 %, *n* = 314). The majority were aged ≥40 years (66.4 %, *n* = 277), ranging from 18 to 60 years. Regarding OH, 95.7 % demonstrated poor knowledge, 65.5 % a positive attitude and 96.6 % poor practices. The Kruskal-Wallis test (*p* < 0.001) showed knowledge scores varied substantially by qualification level. Logistic regression indicated higher qualifications were associated with better knowledge, attitude and practices.

**Conclusion:**

Targeted up-skilling and mandatory obstetric simulation for EC providers in KZN are needed to close knowledge-practice gaps. Despite a positive attitude, EC providers demonstrated poor knowledge and practices in OH management. These shortcomings appear influenced by systemic and contextual barriers and require further investigation. Nevertheless, our findings highlight the importance of sustained, targeted training and EMS support to address critical gaps and improve obstetric outcomes in emergency settings.

## African Relevance

• Obstetric haemorrhage is a leading cause of maternal death in Sub-Saharan Africa yet there is paucity of research regarding prehospital care of these patients.
• Exploring the knowledge, attitude and practices of emergency care providers treatment of obstetric haemorrhage improves insight and adds to the limited body of research.
• The findings of this study provide an opportunity to address gaps through targeted interventions thereby strengthening prehospital care and improving maternal outcomes in KwaZulu-Natal and other similar African settings.

## Introduction

Obstetric haemorrhage (OH) has been described as bleeding that can occur during pregnancy from 20 weeks of gestation through to the end of the puerperium, and is conventionally categorised as antepartum, intrapartum, or postpartum haemorrhage [1,2]. This condition remains a leading cause of maternal mortality worldwide, accounting for approximately 27 % of all maternal deaths annually [3]. Among its subtypes, postpartum haemorrhage (PPH) is a major contributor and is known to cause rapid clinical deterioration if not managed urgently [4]. The World Health Organization (WHO) defines PPH as “blood loss of 500 mL or more within 24 h after birth” [3]. It is estimated that approximately 14 million women experience PPH each year, making it the major direct cause of maternal deaths, particularly in sub-Saharan Africa (SSA) and South Asia [3].

In South Africa (SA), OH is one of the most common direct causes of maternal death [5]. In 2023, a total of 146 OH-related maternal deaths were reported nationwide [5]. The province of KwaZulu-Natal (KZN) accounted for 33 of these deaths, the highest among all provinces highlighting its disproportionate burden. Rurality, transport delays, and limited access to health facilities make timely emergency care critical in KZN [6]. Delays in recognising, diagnosing, and treating OH have been strongly linked to preventable maternal deaths, underscoring the need for timely and effective intervention [1,5,7]. Given SA’s vast geography and the fact that many communities are located far from health facilities, timely emergency response is essential [8]. Emergency Medical Services (EMS) play a critical role in managing obstetric emergencies such as OH and ensuring rapid transport to appropriately equipped health facilities [9]. Emergency Care (EC) providers are front line prehospital professionals that are required to have requisite knowledge and skills to fulfil this role.

Despite the pivotal role of EMS in maternal health emergencies, limited research exists on the preparedness of EC providers to manage OH. Understanding their knowledge, attitudes, and practices (KAP) is essential for identifying training and system-level gaps, strengthening the emergency response to OH, and ultimately improving maternal outcomes.

While some studies in high- and low-income countries have explored EMS response to maternal emergencies, few have specifically examined EC providers' knowledge and practices related to OH, particularly in Africa [[10], [11], [12]]. A recent study by Govender et al. [13] highlighted challenges in emergency care education, including poor training attendance and limited resources as persistent gaps in the EMS system. The current state of EMS may therefore directly affect EC providers’ ability to manage OH and warranted investigation.

The aim of this study therefore was to assess the knowledge, attitude and practices of EC providers regarding the management of OH in KZN.

## Methodology

### Study design and setting

We conducted a cross-sectional survey of EC providers’ knowledge, attitudes, and practices regarding the management of OH in KZN, one of SA’s nine provinces. Although ranked seventh in land area (94,361 km²) [14], KZN is the country’s second-most populous province (≈12.3 million residents) [15]. The provincial EMS operates across 11 health districts and approximately 63 stations, with numbers varying by geography and population density [16].

### Study population and sampling strategy

In SA, there are six categories of EC provider registration, each with varying training durations, academic levels, and clinical scopes of practice (Appendix A). For this study, we grouped participants into three main categories reflecting levels of care and clinical scope: Basic Life Support (BLS), Intermediate Life Support (ILS), and Advanced Life Support (ALS) [17].

*Inclusion criteria* comprised EC providers registered with the Health Professions Council of South Africa (HPCSA) and working on ambulances within the public sector EMS across KZN.

*Exclusion criteria* included EC providers in non-clinical roles (e.g., management, education, or control centre) or not currently practicing on ambulances.

Convenience sampling across the 11 districts yielded strong participation, increasing the calculated minimum sample of 338 to 417 completed questionnaires. District representation was guided by proportional estimates based on EC provider distribution to enhance generalisability.

### Data collection

A 60-item self-administered paper-based questionnaire, developed for this study, covered demographics, knowledge, practice, attitude, preparedness, and recommendations, using multiple-choice, true/false/unsure, Likert-scale, and open-ended formats. Items were based on KAP frameworks in emergency obstetric care and aligned with clinical guidelines. Although no single validated tool was used, content and face validity were confirmed by a multidisciplinary panel of EMS educators, clinical managers, obstetrician and a statistician. The tool was piloted with 16 purposively selected EC providers, resulting in minor wording changes; pilot data were excluded. Data were collected by the principal investigator (PI) at central locations in each of the 11 districts between July 2022 and August 2023, with participants representing multiple ambulance stations within their respective districts.

### KAP scoring

Bloom’s taxonomy is commonly applied to KAP surveys with the following three-tier rubric: good (80–100 %), moderate (60–79 %), and poor (<60 %) performance [18]. Consistent with previous KAP research [19,20], we adopted a modified Bloom threshold of 70 % and dichotomised scores accordingly: respondents scoring ≥70 % were classified as having *good knowledge, positive attitudes, or good practices*, while those scoring <70 % were classified as having *poor knowledge, negative attitudes, or poor practices*. The full scoring rubric is provided in Appendix B. All EC providers (BLS, ILS, and ALS) completed the core KAP questions, which were assessed relative to their HPCSA registration and corresponding level of care to ensure fair evaluation. ALS providers received additional knowledge and practice questions reflecting their broader clinical role, but these were not included in the overall KAP score.

### Data and statistical analysis

Responses were captured in Microsoft Office Excel and analysed in SPSS v29.0 (IBM Corp., Armonk, USA). Descriptive statistics summarised demographics and response distributions, while χ² tests (categorical data) and Mann-Whitney U tests (two-group comparisons) explored univariable associations; knowledge scores across qualification levels were compared with Kruskal-Wallis followed by post-hoc pairwise tests. Multivariable binary-logistic regression adjusted for potential sociodemographic confounders, and exploratory factor analysis of the attitude Likert items identified latent constructs. The questionnaire, a newly developed KAP construct, showed moderate internal consistency (Cronbach’s α = 0.604). While this level of reliability is lower than ideal, it is considered acceptable for a descriptive study with exploratory elements.

### Ethical and health regulatory approval

Ethical approval was obtained from the University of KwaZulu-Natal Biomedical Research Ethics Committee (BREC/00,003,780/2022) and the KwaZulu-Natal Department of Health (KZ/202,205/008). Participation was entirely voluntary, and written informed consent was obtained individually, with assurance that refusal would not affect the participants’ training or institutional relationship. All data were securely stored and accessible only to the PI.

## Results

### Participant characteristics

All participants (*n* = 417) completed the questionnaire. Most were male (75.3 %, *n* = 314), with 24.7 % (*n* = 103) female (Table 1). The majority were aged ≥40 years (66.4 %, *n* = 277), and 33.6 % (*n* = 140) were <40 years. Qualification levels were: BLS providers 35 % (*n* = 146), ILS providers 59.7 % (*n* = 249), and ALS providers 5.3 % (*n* = 22). Most (60.9 %, *n* = 254) graduated from public training institutions (Department of Health Provincial Colleges of Emergency Care and public universities), while 39.1 % (*n* = 163) trained at private colleges or universities. The majority had been registered with the HPCSA for ≥15 years (72.2 %, *n* = 301).

**Table 1:** Demographic characteristics of participants.

Characteristic	n (%)
Age group (years)	
20 – 29	12 (2.9)
30 – 39	128 (30.7)
40 – 49	183 (43.9)
50 – 59	91 (21.8)
60 – 69	3 (0.7)
Biological Sex	
Male	314 (75.3)
Female	103 (24.7)
Level of qualification	
Basic Life Support (BLS)	146 (35.0)
Intermediate Life Support (ILS)	249 (59.7)
Advanced Life Support (ALS)	22 (5.3)
Sector of training (site of training)	
Public training institution	254 (60.9)
Private training institution	163 (39.1)
Years of registration with professional board	
< 15	116 (27.8)
≥ 15	301 (72.2)

### Participant knowledge

**Fig. 1:**
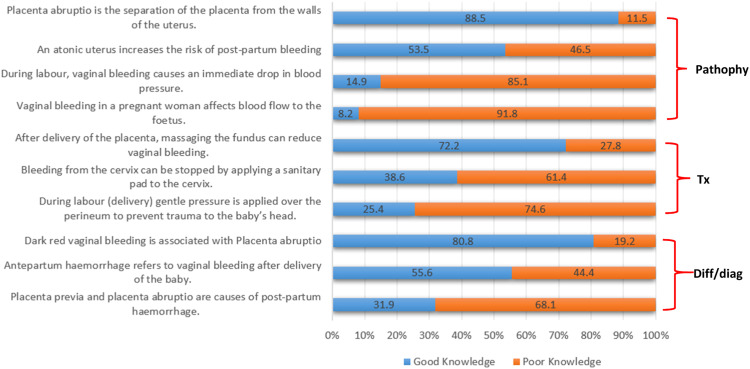
Participants knowledge regarding obstetric haemorrhage. Key: Pathophy = pathophysiology; Tx = treatment; Diff/diag = differential diagnosis.

The majority of participants (95.7 %, *n* = 399) demonstrated poor knowledge regarding the management of OH, with a mean score of 47 % and a median of 50.00 % (IQR: 40.00–60.00) (Fig. 1). Participants were assessed on knowledge of pathophysiology, differential diagnosis, and treatment of OH. Of these, 3 of 4 pathophysiology questions, 2 of 3 differential diagnosis questions, and 2 of 3 treatment questions were poorly answered (Fig. 1).

Knowledge scores differed significantly across qualification levels (Kruskal-Wallis test, *p* < 0.001), suggesting that qualification level had a substantial effect on knowledge. This was supported by pairwise comparisons, which revealed notable differences in mean knowledge scores: BLS providers had the lowest mean score (38.7 %), indicating poor foundational knowledge; ILS providers achieved 50.8 %, showing a marked improvement over the BLS group; and ALS providers attained the highest mean score (58.2 %), reflecting the strongest understanding. However, all scores remained below the KAP threshold of 70 % and were therefore still classified as poor knowledge.

Additionally, of the 22 ALS providers, only 14 were eligible to answer supplementary questions on medications used to treat PPH, as administration of these drugs fell within their individual scope of practice. These responses were scored separately. Among this group, 57.1 % correctly identified Oxytocin as the first-line drug for managing PPH, while only 42.9 % accurately indicated the correct dosage reflecting poor knowledge in this area. These participants were also assessed on the appropriate second-line drug if Oxytocin failed. Tranexamic acid was selected by the majority (71.4 %), indicating good knowledge; however, 92.9 % of these respondents provided an incorrect dosage, again demonstrating poor knowledge.

### Participants attitude

**Fig. 2:**
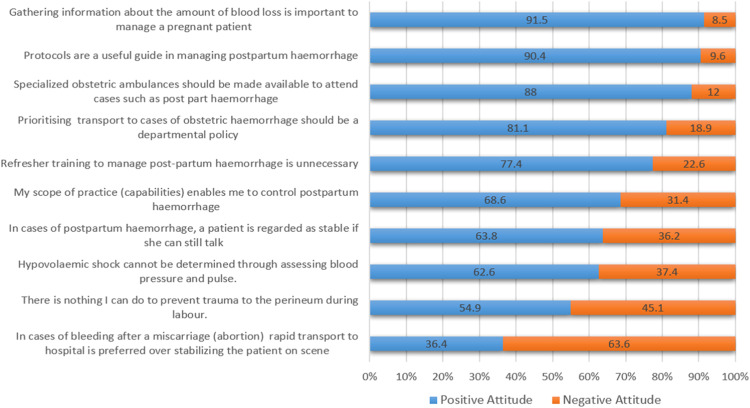
Participants attitude towards management of obstetric haemorrhage.

Majority of the participants (65.5 %, *n* = 273) demonstrated a positive attitude towards management of OH, evidenced by a mean score of 71.5 % and median of 70.00 % (IQR: 60.00–80.00). The responses revealed a general positive disposition, in 9 of the 10 statements regarding OH (Fig. 2).

*Factor analysis* was employed to explore the underlying structure of attitudes held by EC providers towards managing OH. This analysis identified clusters of related attitudes that brought about the following 4 themes: *Protocol Adherence and Emergency Response:* participants supported the concept of a specialized obstetric ambulance the adherence to clinical and departmental guidelines suggesting their preference for structured approaches to manage OH; *Rapid Transport and Shock Management:* participants preferred rapid transport from scene instead of stabilisation of the patient on scene; *Self-Efficacy and Training Attitudes:* participants indicated confidence in skills to manage OH but strongly agreed with continued training; *Misconceptions and Clinical Judgement:* participants displayed inaccurate reasoning, particularly in assessing shock and haemodynamic stability.

### Participants practice

**Fig. 3:**
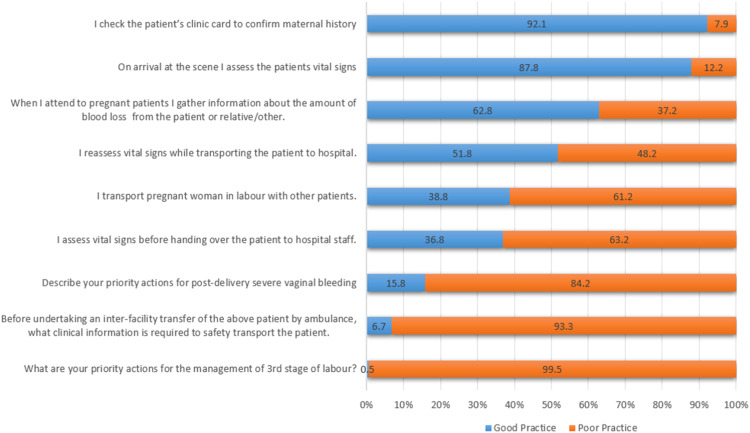
Participants practice towards management of obstetric haemorrhage.

A substantial proportion of participants (96.6 %, *n* = 403) demonstrated poor practice towards the management of OH evidenced through a mean score of 43.7 % and median was 44.4 % (IQR: 33.33–55.56). The responses revealed a general negative disposition, in 5 of the 9 statements regarding the OH (Fig. 3).

### Summary of knowledge, attitude, and practice scores

Table 2 presents a concise summary of KAP scores stratified by key respondent characteristics, including age, type of training institution, medical qualification, and years of experience. A more detailed breakdown is available in Appendix C.

**Table 2:** Knowledge, Attitude and Practice scores regarding obstetric haemorrhage (*N* = 417).

Variable	Category	N (%)	Knowledge Score		Attitude Score		Practice Score	
			Mean	Classification	Mean	Classification	Mean	Classification
Age (years)	20 - 29	12 (2.9)	37.50	Poor Knowledge	65.00	Negative Attitude	44.44	Poor Practice
	30 - 39	128 (30.7)	47.34	Poor Knowledge	71.25	Positive Attitude	43.66	Poor Practice
	40 - 49	183 (43.9)	46.28	Poor Knowledge	70.55	Positive Attitude	43.35	Poor Practice
	50 - 59	91 (21.8)	49.01	Poor Knowledge	74.29	Positive Attitude	43.47	Poor Practice
	60 - 69	3 (0.7)	46.67	Poor Knowledge	80.00	Positive Attitude	66.67	Poor Practice
Highest medical qualification	BLS	146 (35)	38.70	Poor Knowledge	66.51	Negative Attitude	42.24	Poor Practice
	ILS	249 (59.7)	50.80	Poor Knowledge	73.69	Positive Attitude	43.33	Poor Practice
	ALS	22 (5.3	58.18	Poor Knowledge	79.55	Positive Attitude	57.07	Poor Practice
Institution type	Public	254 (60.9)	49.88	Poor Knowledge	73.27	Positive Attitude	44.01	Poor Practice
	Private	163 (39.1)	42.39	Poor Knowledge	68.71	Negative Attitude	43.15	Poor Practice
Years of experience	< 15	116 (27.8)	44.05	Poor Knowledge	70.43	Positive Attitude	46.07	Poor Practice
	≥ 15	301 (72.2)	48.07	Poor Knowledge	71.89	Positive Attitude	42.75	Poor Practice

### Factors associated with knowledge, attitude and practice in managing OH

#### Knowledge

Multivariate analysis showed that providers aged ≥40 years were eight times more likely to demonstrate good knowledge than those <40 years (aOR: 8.17, 95 % CI: 1.40–47.64; *p* = 0.020). In univariate analysis, ILS providers had significantly better knowledge than BLS providers (uOR: 0.15, 95 % CI: 0.05–0.48; *p* = 0.001), and training at a private institution was associated with a three-fold higher odds of good knowledge (uOR: 3.29, 95 % CI: 1.21–8.94; *p* = 0.020). No significant associations were observed for sex, years of registration, or district. Full univariable and multivariable regression coefficients, along with model fit statistics, are provided in Appendix B, Table B1.

#### Attitude

Following multivariate analysis, a higher qualification remained the strongest independent predictor of a positive attitude (OR 1.995, 95 % CI: 1.22–3.26; *p* = 0.006). Private-sector training was also significant (uOR 0.623, 95 % CI: 0.41–0.94; *p* = 0.024), while age ≥ 40 years showed a borderline effect (uOR 1.50, 95 % CI: 0.98–2.29; *p* = 0.060). Sex, years since registration, and district were not significant. Detailed results are provided in Appendix B, Table B2, including model fit statistics.

#### Practice

Qualification exerted the greatest influence on practice. On univariable analysis, ALS providers were 14 times more likely to report good practice (uOR 14.02, 95 % CI: 3.08–63.92; *p* = 0.001), an effect that increased markedly after adjustment (aOR 148.89, 95 % CI: 10.31–2151.26; *p* < 0.001). Providers registered with the HPCSA for ≥15 years also had significantly higher odds of good practice (aOR 32.48, 95 % CI: 1.26–838.21; *p* = 0.036). Age, sex, current qualification registration, and district were not associated with practice. Comprehensive regression results and model fit statistics for the practice domain are presented in Appendix B, Table B3.

## Discussion

In this study, all EC providers completed the same core KAP items, with additional questions for ALS participants excluded from scoring to ensure fair comparison across qualification levels. The results indicate poor knowledge among EC providers regarding the pathophysiology, diagnosis, and treatment of OH, reflected by a mean score of 47 %. For example, 85.1 % did not understand the physiological response to intrapartum bleeding or its effect on blood pressure. Participants also misunderstood signs and conditions related to PPH and antepartum haemorrhage (Fig. 1). These findings suggest that limited pathophysiological knowledge may contribute to suboptimal practice, as 84.2 % demonstrated poor ‘practice’ knowledge inconsistent with HPCSA Clinical Practice Guidelines [21] for managing PPH. Notably, PPH is the only OH subtype covered in detail by these guidelines, which may partly explain the broader knowledge gaps observed across OH.

Similar findings emerged in Australian [10] and Ethiopian [11] studies, which reported paramedics’ weak knowledge in managing obstetric emergencies, aligning with our results. These knowledge gaps reflect barriers identified elsewhere, including irregular attendance [22], infrequent or ineffective obstetric training [12,23], and lower entry-level qualifications [23]. This underscores the need for province-wide up-skilling to enable EC providers to function as true ‘skilled birth attendants’ in the field [24,25].

Despite poor knowledge, EC providers showed a positive attitude, with a mean score of 71.5 %. Although 68.6 % expressed confidence in their skills, 84.2 % (Fig. 3) demonstrated poor practice of shock management in PPH, possibly explaining why 63.5 % preferred rapid transport over stabilization on scene. While this contrasts with HPCSA clinical practice guidelines, it likely reflects practical decision-making within the constraints of provider scope, equipment limitations, and insufficient ALS coverage. Flanagan et al. [10] described this as overconfidence, “a misalignment between actual and self-rated competence” which may lead to substandard clinical care. Conversely, 31.4 % of participants reported low confidence in managing obstetric emergencies, a finding echoed in other studies attributing it to training challenges [10,23,26]. This mix of over- and low confidence mirrors the attitude variability in our study and calls for targeted training interventions. Most participants (77.4 %) valued training, reflecting a positive attitude consistent with other research [10,12,23].

In contrast their positive attitude, EC providers demonstrated poor practice with a mean score of 43.7 % (*p* < 0.001), highlighting risks of negligence, morbidity, and mortality. While adherence to initial vital sign assessments was high (87.8 %), 48.2 % of participants were inconsistent in reassessing vital signs during transport, and 63.2 % failed to conduct final assessments before hospital handover. Poor compliance with vital sign monitoring may stem from operational challenges, such as limited working space in overcrowded ambulances evidenced as 61.2 % of participants reported transporting pregnant women alongside other patients. These findings align with previous studies reporting low compliance with vital sign monitoring [27,28]. Similarly, Griffiths et al. [29] associated missed observations with higher workloads. The mixed practice of transporting pregnant patients with others reflects a resource-driven reality rather than ideal practice [13]. Inadequate monitoring may delay the identification of clinical deterioration, reducing opportunities for early intervention [1,7].

However, poor practice is not solely attributable to individual knowledge or motivation. System-level factors such as EMS protocol quality, dispatch processes, and referral communication may also constrain provider performance. Delays in dispatch, unclear triage protocols, and inconsistent call-centre guidance can undermine timely interventions and adherence to protocols, regardless of provider intent or training.

Moreover, across all three domains; knowledge, attitude and practice; higher medical qualification consistently emerged as the strongest predictor of good performance, an effect that persisted after multivariable adjustment.

Our study found that most EC providers (93.3 %) lacked critical knowledge required to safely take over patients from facility-based providers. Since patient stability is essential for safe transfer, the poor practices noted earlier further emphasize the urgent need for enhanced, qualification-specific training. Variability in provider performance likely reflects these individual knowledge gaps compounded by broader EMS constraints [13].

In summary, EC providers in KZN showed positive attitudes towards managing OH, likely linked to adherence to clinical and departmental guidelines. However, their limited knowledge may have impaired clinical judgement and led to practices misaligned with HPCSA standards. Those with higher qualifications demonstrated stronger knowledge, attitudes, and practices, likely due to structured training and broader clinical exposure. While these gaps reflect provider-level issues, broader system-level barriers such as fragmented dispatch, inconsistent call-centre instructions, weak referral communication, and limited obstetric-specific resources also hinder effective care. A coordinated EMS framework with clear referral pathways, strong communication protocols, and targeted resource support is essential to enable EC providers to function effectively in both field and call-centre settings.

This study is subject to several limitations. The study was set in KZN, limiting generalisability to similar EMS and training contexts. Convenience sampling during training sessions may have introduced selection and social desirability bias. The paper-based tool risked data entry errors, and incomplete or unclear responses were scored as incorrect, potentially lowering practice scores. Although reviewed by experts, the tool lacked formal statistical validation and showed only moderate internal consistency which may limit reliability. Grouping participants with different qualifications could have obscured variations in training and scope of practice. Small subgroup sizes may have contributed to unstable regression estimates, seen in wide confidence intervals and large adjusted odds ratios. Nevertheless, strengths included high participation, wide district coverage, and multivariable analysis to explore influencing factors.

## Conclusion

This study reveals that despite EC providers positive attitude, they demonstrated limited knowledge and inconsistent practices in managing OH. Addressing these limitations requires urgent investment in qualification-specific training and system-wide improvements. Future research should explore innovative strategies such as high-fidelity simulations and real-time teleconsultation to enhance provider readiness. A review of EMS training programmes, particularly within the KZN College of Emergency Care, in alignment with the Department of Health’s Essential Steps in the Management of Obstetric Emergencies (ESMOE) initiative, may support more effective integration of national guidelines into practice. Strengthening maternal emergency care in the province will require a coordinated, multi-stakeholder response that goes beyond individual provider performance.

## Dissemination of results

The result of this study will be presented to the National School of Government online research presentations, KZN Provincial Emergency Medical Service Committee and the KZN College of Emergency Medical Care.

## Declaration

This study formed part of a thesis requirement for a current doctoral study regarding the management of OH by Emergency Care Providers in the KwaZulu-Natal. The principal investigator received a scholarship from the College of Health Sciences at the University of KwaZulu-Natal.

## Dissemination of results

The result of this study will be presented to the National School of Government online research presentations, KZN Provincial Emergency Medical Service Committee and the KZN College of Emergency Medical Care.

## Declaration of generative AI use

The authors did not employ generative AI tools in the writing of this manuscript.

## CRediT authorship contribution statement

**S Govender:** Conceptualization, Methodology, Investigation, Resources, Writing – original draft. **OP Khaliq:** Project administration, Visualization, Writing – review & editing. **T Abel:** Data curation, Validation, Writing – review & editing. **J Moodley:** Supervision, Validation, Writing – review & editing.

## Declaration of competing interest

There is no conflict of interest to declare.

## Appendix Supplementary materials

Image, application 1
