# Supplementary material for: Knowledge, attitude and practice of emergency care providers on obstetric haemorrhage in KwaZulu-Natal, South Africa: A cross-sectional study
Source: Afr J Emerg Med. 2025 Sep 27;15(4):100909. doi: 10.1016/j.afjem.2025.100909 (PMC12510202; doi:10.1016/j.afjem.2025.100909)
Supplement: Supplementary file 1 [file mmc1.docx]

**Appendix A**

*Study Population and Sampling Strategy*

The target population comprised all operational Emergency Care (EC) providers employed in the public EMS sector across KwaZulu-Natal (KZN) and registered with the Health Professions Council of South Africa (HPCSA). In South Africa, six categories of registration exist, each associated with varying training durations, academic levels, and clinical scopes of practice. These include Basic Ambulance Assistant (BAA), Ambulance Emergency Assistant (AEA), Critical Care Assistant (CCA), Emergency Care Assistant (ECA), Emergency Care Technician (ECT), and Emergency Care Practitioner (ECPs).

For the purpose of this study, participants were grouped into three main categories reflecting general levels of care and clinical scope:

- Basic Life Support (BLS) – appears in the “Regulations relating to the Standards for Emergency Medical Services, 2022” - comprising BAA qualifications (supervised practice) and are not permitted to manage patients independently without oversight from a higher-qualified provider.
- Intermediate Life Support (ILS) – appears in the “Regulations relating to the Standards for Emergency Medical Services, 2022” - comprising AEA (independent practice). The ECA has been included in this ILS category. The ECA has a greater scope of practice than BLS providers but less than ALS, making it better suited for inclusion in ILS for this study.
- Advanced Life Support (ALS) – appears in the “Regulations relating to the Standards for Emergency Medical Services, 2022” - comprising CCA, ECT, and ECP qualifications (independent practice)

Emergency Care Technicians (ECTs) and Emergency Care Practitioners (ECPs) were grouped under ALS due to their expanded scopes of practice, alignment with advanced prehospital care as expressed in the “Regulations Relating Standards for Emergency Medical Services”:

"Advanced Life Support (ALS)" means a level of care provided within the Paramedic, Emergency Care Technician or Emergency Care Practitioner scope of practice as determined by the Health Professions Council of South Africa in terms of the Health Professions Act, 1974 (Act No. 56 of 1974)

We acknowledge that it simplifies a complex qualification structure and may limit nuanced interpretation of some findings. This limitation is noted in the discussion.

**Appendix B**

*KAP scoring*

For the KAP assessment, the Bloom’s cut-off points are often used and are as follow: 80%–100% (good KAP), 60%–79% (moderate KAP) and less than 60% (poor KAP).^11-13^ In this study, a modified Bloom’s cut-off value of 70% was used to categorise participants’ KAP into two levels: Participants scoring 70% or above in the assessment are categorized as having good knowledge, positive attitudes, or good practices.

Participants scoring below 70% are categorized as having poor knowledge, negative attitudes, or poor practices.

For the knowledge component, there were 10 questions in the format of: ‘true, unsure, false’. A correct answer was given 1 point while an incorrect or ‘unsure’ answer was given zero. A score within the range of 0 to 6 indicated poor knowledge, whereas a score from 7 to 10 indicated good knowledge.

For the attitude component, there were 10 questions needing responses from a 5 point-point Likert scale (strongly disagree; disagree; unsure; agree; strongly agree). A positive attitude was given one point and a negative attitude including ‘unsure’ was given a zero. A score within the range of 0 to 6 indicated negative attitude, whereas a score from 7 to 10 indicated a positive attitude.

Also, regarding the attitude component, for certain items, such as *“In cases of bleeding after a miscarriage, rapid transport to hospital is preferred over stabilizing the patient on scene,”* a **disagree** response was scored as a **positive attitude**, as it aligns with the HPCSA Clinical Practice Guidelines that emphasize stabilizing patients before transport when feasible. This scoring was applied consistently based on alignment with clinical best practices.

For the practice component, there were 9 questions all together. There were 6 questions in the format of: ‘Often, sometimes, rarely, no’ and 3 open ended (short narrative) questions. Good practice was given one point and poor practice was given a zero. The open-ended questions were given zero if it was not answered, incompletely or partially answered. A score within the range of 0 to 6 indicated poor practice, whereas a score from 7 to 9 indicated good practice. Practice responses were assessed in relation to the participant’s qualification level and corresponding HPCSA scope of practice to ensure fair evaluation across all provider levels.

**Appendix C**


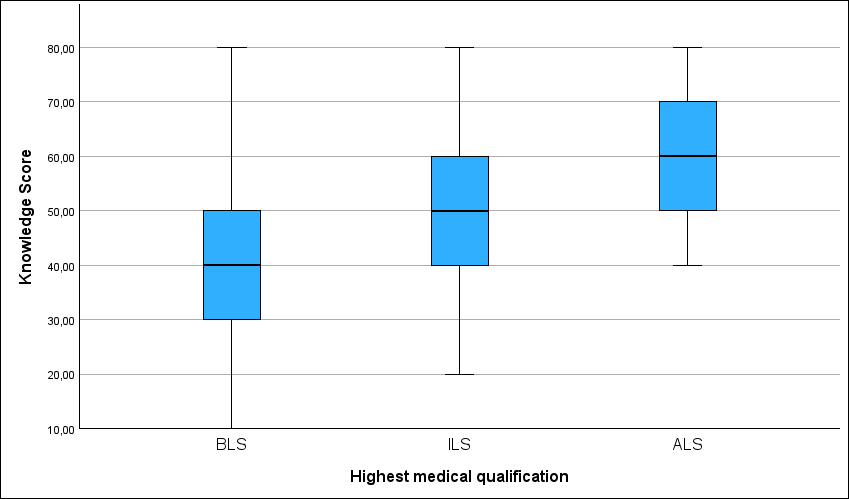


**Fig. C1 Participants mean knowledge score by qualification**


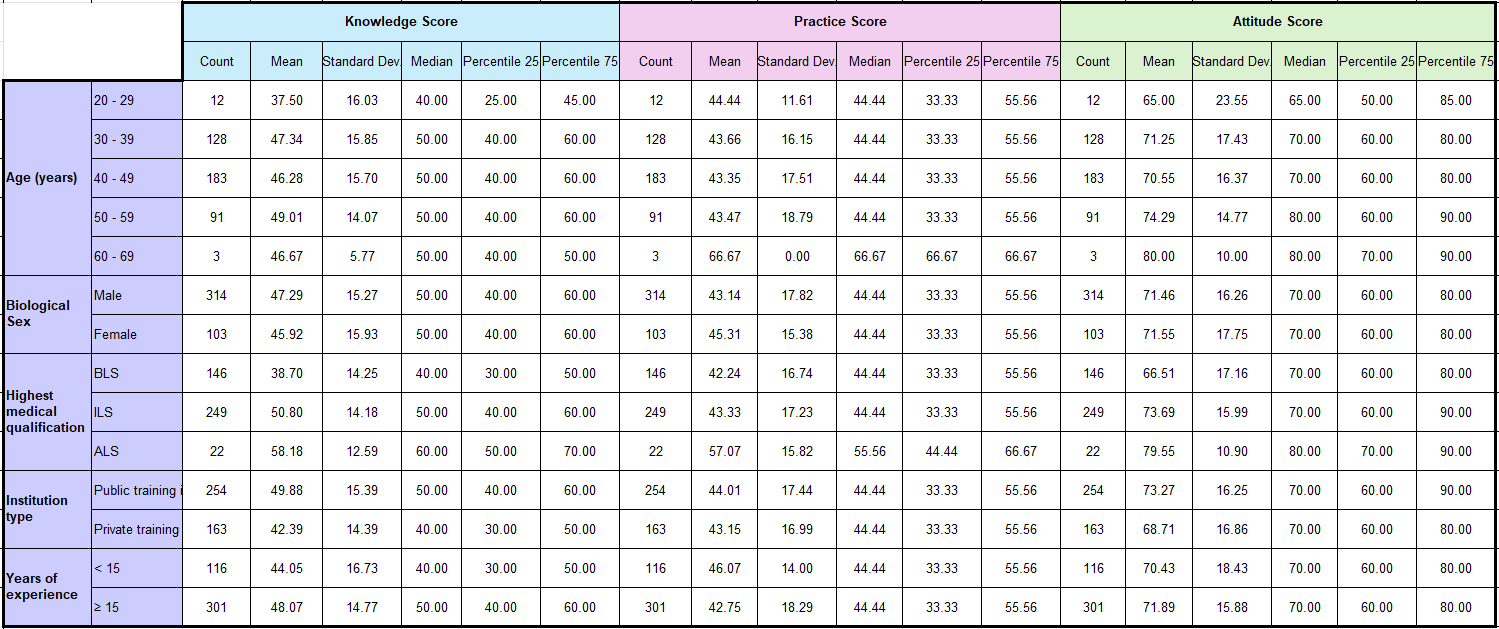


**Fig. C2 Distribution of Knowledge, Attitude, and Practice (KAP) Scores by Respondent Characteristics**

**Table C1. Logistic regression analysis of biographical factors associated with knowledge (n=417)**

|  | **Univariable analysis** | | **Multivariable analysis** | |
| --- | --- | --- | --- | --- |
| **Variable** | **OR (95% CI)** | **p-value** | **OR (95% CI)** | **p-value** |
| Age (≥40 years compared to < 40 years) | **4.23** (0.959 - 18.663) | 0.057 | **8.171** (1.401 - 47.643) | **0.020** |
| Biological Sex (Male compared to Female) | 1.557 (0.569 - 4.258) | 0.389 | 1.277 (0.385 - 4.238) | 0.690 |
| What is your highest medical qualification? |  | **0.005** |  | 0.764 |
| Highest Medical Qualification (ILS compared to BLS) | 0.154 (0.05 - 0.477) | **0.001** | 0.241 (0.052 - 1.107) | 0.067 |
| Highest Medical Qualification (ALS compared to BLS) |  | 0.998 |  |  |
| Where Medical Qualification Attained (Private compared to Public Institution) | **3.285** (1.208 - 8.935) | **0.020** | **1.483** (0.429 - 5.124) | 0.533 |
| HPCSA Registration Duration (≥15 years compared to <15 years) | 0.761 (0.279 - 2.078) | 0.594 | 0.484 (0.117 - 1.993) | 0.315 |
| Current Qualification Registration Duration (≥10 years compared to <10 years) | **6.634** (0.873 - 50.441) | 0.068 | **2.476** (0.237 - 25.862) | 0.449 |
| Municipal District (King Cetshwayo compared to eThekwini) | 1.094 (0.951 - 1.259) | 0.207 |  | 0.778 |

*Odds Ratio (OR)*: An OR > 1 indicates increased odds of the outcome occurring in the comparison group, whereas an OR < 1 suggests decreased odds. An OR = 1 implies no effect.

*95% Confidence Interval (CI)*: Represents the range within which the true OR is expected to lie with 95% certainty. A wider interval suggests greater uncertainty in the estimate.

*P-value*: Indicates statistical significance. p < 0.05 denotes statistical significance, p < 0.01 represents strong significance, and p < 0.001 indicates very strong significance.

The logistic regression model assessing predictors of knowledge demonstrated acceptable fit. The Hosmer-Lemeshow test was non-significant (χ² = 4.367, df = 8, p = 0.823), indicating good calibration. The -2 Log-Likelihood was 67.219, and the Nagelkerke R² was 0.420, indicating moderate explanatory power. The model correctly classified 95.7% of responses.

**Table C2.** **Logistic regression analysis of biographical factors associated with attitude (n=417)**

|  | **Univariable analysis** | | **Multivariable analysis** | |
| --- | --- | --- | --- | --- |
| **Variable** | **OR (95% CI)** | **p-value** | **OR (95% CI)** | **p-value** |
| Age (≥40 years compared to < 40 years)) | 1.5 (0.984 - 2.287) | 0.060 | 1.451 (0.846 - 2.488) | 0.176 |
| Biological Sex (Male compared to Female) | 1.033 (0.646 - 1.652) | 0.892 | 1.107 (0.669 - 1.831) | 0.693 |
| What is your highest medical qualification? |  | 0.001 | 1.995 (1.22 - 3.263) | 0.006 |
| Highest Medical Qualification (ILS compared to BLS) | 1.878 (1.23 - 2.867) | 0.004 |  |  |
| Highest Medical Qualification (ALS compared to BLS) | 8.25 (1.86 - 36.593) | 0.005 |  |  |
| Where Medical Qualification Attained (Private compared to Public Institution) | 0.623 (0.413 - 0.94) | 0.024 | 0.91 (0.545 - 1.518) | 0.717 |
| HPCSA Registration Duration (≥15 years compared to <15 years) | 1.361 (0.874 - 2.121) | 0.173 | 1.052 (0.579 - 1.911) | 0.868 |
| Current Qualification Registration Duration (≥10 years compared to <10 years) | 0.896 (0.567 - 1.417) | 0.640 | 1.084 (0.581 - 2.02) | 0.801 |
| Municipal District |  | 0.282 |  | 0.438 |

Odds Ratio (OR), 95% Confidence Interval (CI), P-value

The model for attitude indicated acceptable model fit. The Hosmer-Lemeshow test was non-significant (χ² = 5.592, df = 8, p = 0.693), suggesting good calibration. The -2 Log-Likelihood was 517.612, and the Nagelkerke R² was 0.083, reflecting limited explanatory capacity. The model correctly classified 65.5% of cases.

**Table C3. Logistic regression analysis of biographical factors associated with practice (n=417)**

|  | **Univariable analysis** | | **Multivariable analysis** | |
| --- | --- | --- | --- | --- |
| **Variable** | **OR (95% CI)** | **p-value** | **OR (95% CI)** | **p-value** |
| Age (≥40 years compared to < 40 years) | 1.888 (0.518 - 6.882) | 0.335 | 0.734 (0.141 - 3.818) | 0.713 |
| Biological Sex (Male compared to Female) | 0.826 (0.226 - 3.021) | 0.773 | 1.306 (0.293 - 5.822) | 0.727 |
| What is your highest medical qualification? |  | < 0.001 |  | < 0.001 |
| Highest Medical Qualification (ILS compared to BLS) | 1.177 (0.29 - 4.779) | 0.82 | 2.886 (0.402 - 20.738) | 0.292 |
| Highest Medical Qualification (ALS compared to BLS) | 14.02 (3.075 - 63.916) | 0.001 | 148.889 (10.305 - 2151.259) | < 0.001 |
| Where Medical Qualification Attained (Private compared to Public Institution) | 1.175 (0.4 - 3.451) | 0.769 | 3.685 (0.674 - 20.151) | 0.132 |
| HPCSA Registration Duration (≥15 years compared to <15 years) | 5.191 (0.671 - 40.138) | 0.115 | 32.479 (1.259 - 838.211) | 0.036 |
| Current Qualification Registration Duration (≥10 years compared to <10 years) | 1.377 (0.377 - 5.027) | 0.629 | 0.744 (0.106 - 5.223) | 0.766 |
| Municipal District |  | 0.999 |  | 0.994 |

Odds Ratio (OR), 95% Confidence Interval (CI), P-value

The logistic regression model for practice demonstrated robust fit. The Hosmer-Lemeshow test was non-significant (χ² = 6.393, df = 8, p = 0.604), indicating that the model's predictions matched observed outcomes. The -2 Log-Likelihood was 63.176, and the Nagelkerke R² was 0.682, suggesting strong explanatory power. The overall classification accuracy was 97.9%.
